# A Twisting Tale of Infective Endocarditis

**DOI:** 10.7759/cureus.6116

**Published:** 2019-11-10

**Authors:** Abeera Akram, Uzochukwu Ibe, Ahmed Kazi

**Affiliations:** 1 Internal Medicine, Saint Mary's Hospital, Waterbury, USA; 2 Cardiology, Danbury Hospital, Danbury, USA

**Keywords:** enterococcus endocarditis, torsades de pointes

## Abstract

We present a case of a unique complication of enterococcus endocarditis in an elderly man with a cardiac pacemaker who presented with low-grade fever and cough. He had no history of IV drug use. Blood cultures were positive for Enterococcus faecalis, Both trans-thoracic echo and trans-esophageal echo showed vegetation on the aortic valve. He was discharged on adequate antibiotic coverage but later presented again with near syncope. On cardiac monitoring, he was found to have episodes of torsades. Unlike heart failure or peri-valvular abscess, torsades is a rare complication of endocarditis. We aim to present a case report of a rare complication of infective endocarditis (IE) which if not identified timely, can lead to lethal outcomes. Unfortunately, our patient did not survive, but we learnt that though rare, we should always anticipate rhythm problems such as torsades as complications of endocarditis and should promptly treat with magnesium and antiarrhythmic drugs such as lidocaine if needed.

## Introduction

Torsades de pointes (TDP), translated as "twisting of peaks" is a polymorphic ventricular tachycardia that can lead to sudden death. This report aims to highlight a case of infective endocarditis (IE), which led to a rare complication in the form of TDP. Commonly after IE, patients present either with heart failure, peri-valvular abscess or pericarditis, but our patient, despite getting adequate antibiotics not only presented with acute heart failure but also TDP. Unfortunately he did not survive but gave us a reason to ponder that rhythm abnormalities should always be kept in mind and require prompt treatment if we are dealing with complications of IE.

## Case presentation

**Figure 1:**
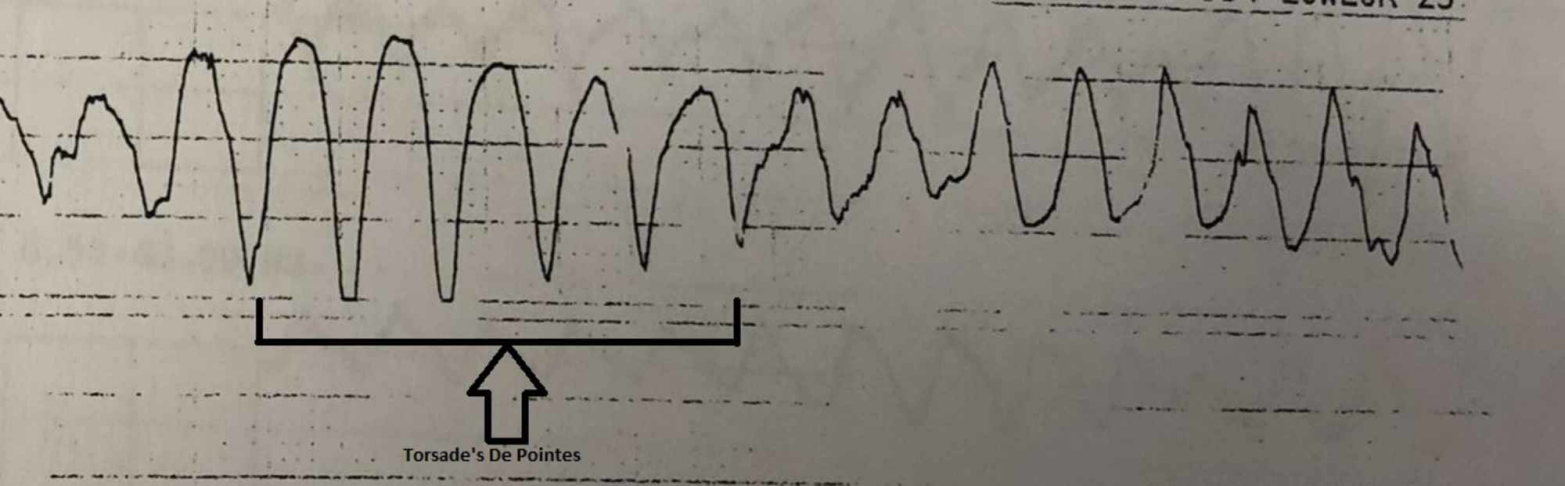
ECG strip showing torsades de pointes. ECG: electrocardiogram

A 75-year-old male with past medical history of paroxysmal atrial fibrillation status post cardiac pacemaker, hypertension presented to urgent care clinic with several weeks of low-grade fever, cough, and shortness of breath. He was discharged on augmentin for possible pneumonia as there was a questionable retrocardiac infiltrate on chest X-ray. Three days later, he presented in the ED with complaints of acute dyspnea in addition to fever, cough, and fatigue for one month. His initial vitals were blood pressure 127/54 mmHg, pulse rate 77 beats per minute, respiratory rate 20 breaths per minute, temperature 100.3°C, and an oxygen saturation of 100% on room air. He was alert and oriented and physical examination was significant for an unexplained murmur. He was admitted on medical floors; lab workup was sent including blood cultures and urine cultures. The infectious disease department was consulted for fever of unknown origin and augmentin was discontinued. The initial differential diagnosis included endocarditis, occult malignancy, and recurrent pulmonary emboli (patient's son recently sustained a pulmonary embolism). On day two of admission, 1/2 sets of blood cultures were positive for *Enterococcus faecalis*, then two more subsequent sets were positive. The patient was started on ampicillin plus ceftriaxone, gentamicin for six weeks. The organism was later found to be sensitive to rifampin, so this agent was also added. Trans-thoracic echocardiogram was done which showed severe aortic valve regurgitation. Trans-esophageal echocardiogram was also done which showed a 1 cm x 1 cm mass on the aortic valve. When the bacteremia resolved, he was discharged on ampicillin, ceftriaxone, rifampin for six weeks, and gentamicin for two weeks. Two days after discharge, the patient presented again to the ED with a near syncopal event and worsening shortness of breath. Lab work was significant for hypomagnesemia. Brain-natriuretic peptide (BNP) was also elevated and chest radiograph showed small bilateral pleural effusions suggestive of acute pulmonary edema/heart failure. He was treated with Lasix. The following day, the patient had an episode of self-limiting torsades (Figure 1).

He was given IV magnesium and was started on amiodarone, which was later changed to a lidocaine drip. At this point, he was transferred to the critical care unit and cardiothoracic surgery was consulted for aortic valve replacement and a coronary angiogram was scheduled. Cardiac catheterization showed multi-vessel disease. The patient underwent aortic valve replacement, coronary artery bypass graft for triple-vessel disease, ligation of left atrial appendage, and evacuation of the bilateral pleural effusions. His post-op course was complicated by increased chest tube drainage for which subsequent exploration was done. The patient was given prothrombin complex concentrate and blood products. He required continued intubation and vasopressor support for hemodynamic stability. Eventually the patient was weaned off the vasopressor support, urine output improved but he developed elevated liver enzymes, probably due to hypoperfusion to the liver for an ultrasound did not reveal any biliary obstruction. After a day he again developed hypotension and was restarted on vasopressors and laboratory assessment revealed leukocytosis. Surgery and gastroenterology were consulted for pancolitis evaluation. No intervention was recommended as patient was not stable enough; he was started on fluconazole for Candida (sputum and urine culture grew Candida) and flagyl for possible pancolitis. The patient started to have worsening liver function tests; cholecystostomy tube was placed. The patient’s hospital course continued to decline and realizing his poor prognosis, the family decided to withdraw all care. He subsequently died of cardiopulmonary arrest.

## Discussion

Staphylococci, including *Staphylococcus aureus* and coagulase-negative Staphylococcus, are the most common pathogens responsible for cardiovascular implantable device infection [1]. Interestingly our patient had *E. faecalis*. *Enterococcus faecalis* IE is a disease of increasing importance, with more patients infected, increasing frequency of health-associated infections and increasing incidence of antimicrobial resistance. Usually the typical presentation, as it was in our patient, is sub-acute fever, malaise, generalized aches, difficult to distinguish from other more common diseases. [2] It is a severe infection that still has an in-hospital mortality of 15%-20% and a one year mortality of approximately 25%-30% [3]. To establish the diagnosis in addition to blood cultures, transthoracic and transesophageal echocardiographic evaluations are of paramount importance. At the moment, the most commonly employed treatment pair is ampicillin with either ceftriaxone or gentamicin [1]. Infective endocarditis is associated with a broad range of complications depending on factors, which include the type of pathogen, duration of illness, and underlying comorbidities. The most common complications include cardiac, neurological, renal, musculoskeletal, pulmonary, septic embolization, and mycotic aneurysms. Among these, cardiac complications are the most common. Heart failure is the most common cause of death due to IE. Other cardiac complications involve perivalvular abscess, pericarditis, and intracardiac fistula. Usually patient who presents with new conduction abnormalities on electrocardiogram (ECG) has a suspicion of perivalvular abscess [4].

Our patient also had acute heart failure but surprisingly conduction abnormalities were also found on his ECG. In a study of patients with endocarditis, performed at the Duke Medical Center, 26% had conduction abnormalities. The most common type identified was first degree atrio-ventricular but our patient had TDP [5]. Torsades de pointes is a form of a polymorphic ventricular arrhythmia associated with QTc interval prolongation. A few cases of IE complicated by TDP have been reported. A literature review of pubmed was done. We found cases of QT interval prolongation complicated with TDP in a prosthetic mitral valve endocarditis [6], in an IV drug abuse-associated IE [7], in a prosthetic mitral valve Brucella endocarditis complicated with TDP [8]. Interestingly our patient had a unique presentation: he was not an IV drug abuser, had no prosthetic valve, and the infective pathogen was enterococcus.

In such cases, magnesium sulfate is the treatment of choice for TDP and lidocaine can also be used. Serum potassium should be checked and maintained at a high normal level. A patient can also benefit from temporary transvenous pacing; if all the treatment is ineffective, implanted defibrillator should be carried out.

## Conclusions

Various studies suggest that patients who develop conduction abnormalities during their hospitalization stay have poor prognosis, so we should be vigilant for TDP in patients with endocarditis because while it is rare it is highly lethal. Strategies to prevent IE, improved diagnostics, and optimized treatment will be necessary to improve the overall prognosis.
